# SOX7 expression is critically required in FLK1-expressing cells for vasculogenesis and angiogenesis during mouse embryonic development

**DOI:** 10.1016/j.mod.2017.05.004

**Published:** 2017-08

**Authors:** Andrew J. Lilly, Andrzej Mazan, Daryl A. Scott, Georges Lacaud, Valerie Kouskoff

**Affiliations:** a Cancer Research UK Manchester Institute, The University of Manchester, Wilmslow road, M20 4BX, UK; b Department of Molecular and Human Genetics, Baylor College of Medicine, One Baylor Plaza, BCM227, Houston, TX 77030, USA; c Division of Developmental Biology and Medicine, The University of Manchester, Michael Smith Building, Oxford Road, Manchester M13 9PT, UK

**Keywords:** Vascular development, SOXF, Endothelium

## Abstract

The transcriptional program that regulates the differentiation of endothelial precursor cells into a highly organized vascular network is still poorly understood. Here we explore the role of SOX7 during this process, performing a detailed analysis of the vascular defects resulting from either a complete deficiency in *Sox7* expression or from the conditional deletion of *Sox7* in FLK1-expressing cells. We analysed the consequence of *Sox7* deficiency from E7.5 onward to determine from which stage of development the effect of *Sox7* deficiency can be observed. We show that while *Sox7* is expressed at the onset of endothelial specification from mesoderm, *Sox7* deficiency does not impact the emergence of the first endothelial progenitors. However, by E8.5, clear signs of defective vascular development are already observed with the presence of highly unorganised endothelial cords rather than distinct paired dorsal aorta. By E10.5, both *Sox7* complete knockout and FLK1-specific deletion of *Sox7* lead to widespread vascular defects. In contrast, while SOX7 is expressed in the earliest specified blood progenitors, the VAV-specific deletion of *Sox7* does not affect the hematopoietic system. Together, our data reveal the unique role of SOX7 in vasculogenesis and angiogenesis during embryonic development.

## Highlights

• *Sox7* is expressed at the onset of endothelial specification from mesoderm.
• *Sox7* deficiency does not impact the emergence of the first endothelial progenitors.
• *Sox7* complete knockout and FLK1-specific deletion lead to widespread vascular defects.
• VAV-specific deletion of *Sox7* does not affect the hematopoietic system.
• Unique role of SOX7 in vasculogenesis and angiogenesis during embryonic development

## 1 Introduction

The development of the vascular system involves a complex array of processes necessary to regulate the dynamic nature of the emerging vascular network. During development, the first blood vessels form in the extra-embryonic yolk sac *via* vasculogenesis, which initiates following the formation of blood islands from mesodermal progenitors (Ferkowicz and Yoder, 2005). Cells on the inside of the blood islands differentiate into blood cells, whereas cells on the outside differentiate into endothelial precursor cells (EPCs), which migrate and associate to form a primitive vascular plexus (Herbert and Stainier, 2011). In the embryo proper, EPCs migrate to form endothelial chords that differentiate into the major arteries and veins (De Val and Black, 2009). The primitive extra and intra-embryonic vascular network subsequently undergoes angiogenesis involving the remodelling and expansion of blood vessels resulting in the formation of a hierarchically organized vascular network (Risau and Flamme, 1995). The transcriptional network regulating the identity and behaviour of EPCs involved in vascular development is extremely complex and remains poorly understood.

The *Sox* family of genes encodes a group of transcription factors that all share a high mobility group (HMG) DNA binding domain and recognise the AACAAT consensus sequence (Schepers et al., 2002). The SOX F subgroup contains SOX7, SOX17 and SOX18, and a growing body of evidence indicates that they have important roles in cardiovascular development (Francois et al., 2010, Lilly et al., 2017). However, SOX17 has pleiotropic functions and regulates a variety of processes including: definitive endoderm specification (Kanai-Azuma et al., 2002), fetal hematopoietic stem cell proliferation (Kim et al., 2007), oligodendrocyte development (Sohn et al., 2006) and arterial specification during cardiovascular development (Corada et al., 2013). The role of SOX18 appears to be more restricted with deficiency in this factor leading to specific defects in lymphangiogenesis (Francois et al., 2008). In contrast, the role and function of SOX7 is still poorly defined. SOX7 is expressed in primitive endoderm (Futaki et al., 2004, Murakami et al., 2004) and in endothelial cells at various stages of vascular development. These include the mesodermal masses that give rise to blood islands in gastrulating embryos (Gandillet et al., 2009), and the vascular endothelial cells of the dorsal aorta, intersomitic vessels and cardinal veins in more developed embryos (Hosking et al., 2009, Kim et al., 2016, Takash et al., 2001). Gross morphological examination of *Sox7*^*−/−*^ mouse embryos suggests potential vascular defects (Wat et al., 2012); more recently, it was shown that the conditional deletion of *Sox7* in *Tie2* expressing endothelial cells results in branching and sprouting angiogenic defects at E10.5 (Kim et al., 2016). Despite these recent advances, a comprehensive analysis of the developing vascular network encompassing both vasculogenic and angiogenic processes in SOX7 deficient embryos has not yet been undertaken.

Here, we performed a detailed analysis of the vascular defects resulting from either a complete deficiency in *Sox7* expression or from the conditional deletion of *Sox7* in FLK1-expressing cells.

## 2 Materials and methods

### 2.1 ESC culture and differentiation

Embryonic stem cells (ESCs) were cultured and differentiated as previously described (Sroczynska et al., 2009). Embryoid bodies (EBs) were routinely maintained up to day 3, and FLK1^+^ cells were isolated and cultured as previously described (Fehling et al., 2003, Lancrin et al., 2009).

### 2.2 Generation of Sox7 knockout mouse lines

Targeted *Sox7* ESC clone B9 (International Knockout Mouse Consortium) was injected into mouse blastocysts. Resultant chimaeras were crossed with C57BL/6 mice. Subsequent generations were crossed with PGK-Cre mice to excise the neomycin cassette and exon 2 of the *Sox7* gene, resulting in the generation of a LacZ-tagged null allele (*Sox7*^LacZ/WT^). Alternatively to generate the *Sox7*-floxed allele, mice were crossed with an actin-FLP transgenic line resulting in the excision of both IRES-LacZ and neomycin cassettes that are flanked by FRT site (International Knockout Mouse Consortium). After eight backcrosses on C57BL/6, mice were either inter-crossed to generate *Sox7*^*fl/fl*^ or crossed with *Flk1-cre* (Motoike et al., 2003) or *Vav-Cre* (de Boer et al., 2003) transgenic lines to excise in a tissue specific manner the exon 2 of *Sox7* that is flanked by LoxP sites.

### 2.3 Timed matings

Timed matings were set up between: heterozygous male and female *Sox7*^*LacZ/WT*^ mice, heterozygous *Sox7*^*fl/wt*^ *Flk1-Cre* male and *Sox7*^*fl/fl*^ or *Sox7*^*fl/−*^ female mice. The morning of vaginal plug detection was embryonic day (E) 0.5. All animal work was performed under regulation governed by the Home Office Legislation under the Animal Scientific Procedures Act (ASPA) 1986.

### 2.4 QRT-PCR

Total RNA was isolated using Rneasy Mini/Micro plus Kit (Qiagen), and 2 μg of which was used to generate cDNA using the Omniscript reverse transcriptase kit (Qiagen), according to the manufacturer's instructions. Real time PCR were performed on an ABI 7900 system (Applied Biosystems) using the Exiqon universal probe library (Roche). Gene expression was calculated relative to β-actin using the ΔΔCt method.

### 2.5 Whole mount and section staining

Embryos were stained using a rat anti-mouse CD31 antibody (1:500) (BD biosciences; 553,370) and a goat anti-rat AF555 secondary antibody (1:1000) (Invitrogen) as previously described (Yokomizo et al., 2012). *Z*-stack images were taken using a two-photon confocal microscope with a 5 × objective (Leica). E10.5 embryo sections were stained as previously described (Thambyrajah et al., 2016) using a goat anti-SOX7 antibody (1:200) (R&D systems; AF2766) and a donkey anti-goat AF647 antibody (1:2000) (Invitrogen). Subsequently, embryos were stained with a rat anti-cKit antibody (1:1000) (BD biosciences; 553,868), and a rabbit anti-pan-RUNX antibody (1:1000) (Abcam; ab92336) before staining with a goat anti-rat AF488 and a goat anti-rabbit antibody (both 1:2000) (both Invitrogen). Yolk sacs were isolated and flat mounted with DAPI as previously described (Frame et al., 2016) before imaging. Specific labelling of primary antibodies was determined by comparison with no primary antibody stained controls.

### 2.6 Flow cytometry

Cells were disaggregated by trypsinisation, and incubated with combinations of conjugated monoclonal antibodies on ice. Analyses were performed on a BD LSRII (BD Biosciences). Data were analysed with FlowJo (TreeStar), gating first on the forward scatter *versus* side scatter to exclude non-viable cells.

### 2.7 Statistical analyses

Sample sizes were chosen based on previous experimental experience. Student's *t*-test was used to assess the differences between two populations in embryo experiments. * *P*-value < 0.05, **P-value < 0.01, *** *P*-value < 0.001.

## 3 Results and discussion

### 3.1 SOX7 is expressed in EPCs at the onset of endothelial specification from mesoderm

**Fig. 1:**
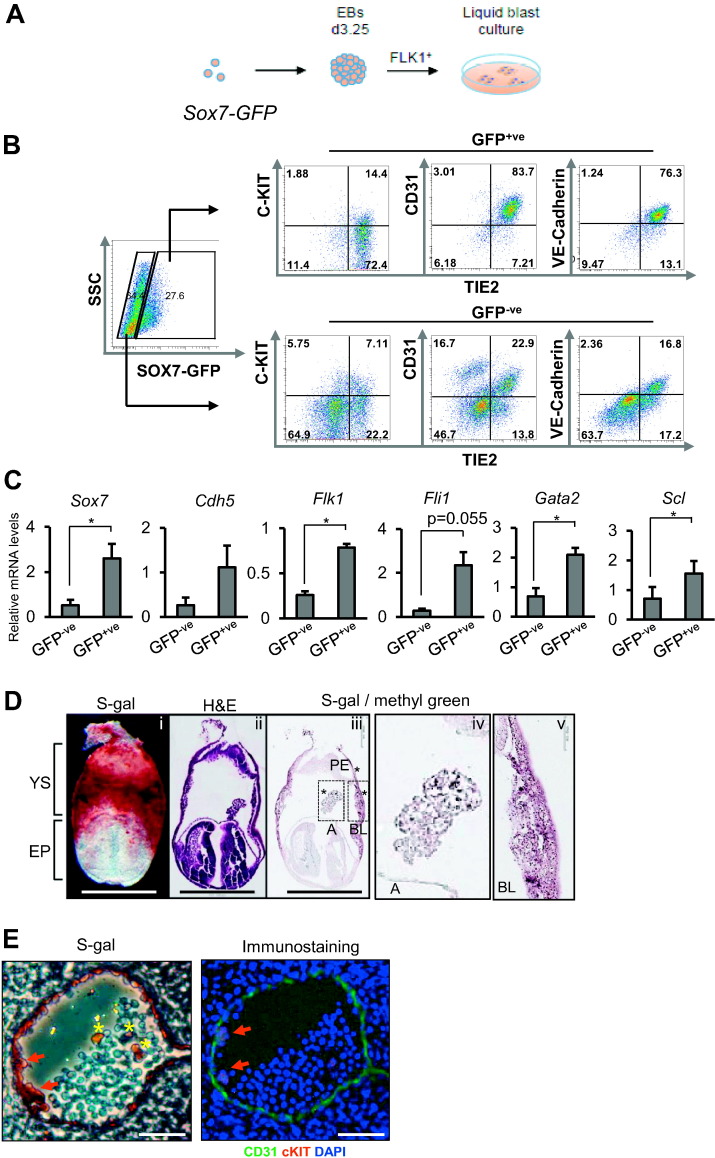
SOX7 is expressed at the onset of endothelial differentiation from mesodermal precursors. (A) FLK1^+^ cells were sorted from day 3.25 *Sox7-Gfp* embryoid bodies (EBs) and cultured in 2D culture. (B) Flow cytometry analysis of SOX7-GFP^+^ and SOX-GFP^−^ fractions at day 2 of culture. Data are representative of 3 independent experiments. (C) QRT-PCR analysis for the expression of the indicated genes in sorted SOX7-GFP^+^ and SOX7-GFP^−^ fractions at day 2 of the culture. Error bars indicate ± SEM (n = 3 independent experiments), Student's paired two-tailed *t*-test. (D) E7.5 *Sox7*^*LacZ/WT*^ embryos: (i) whole mount S-gal staining, (ii) hematoxylin and eosin (H&E) staining on section, (iii) S-gal and methyl green staining on section, (iv) close-up image of allantois from the S-gal staining, (v) close-up image of blood island from the S-gal staining. YS: yolk sac, EP: embryo proper, A: allantois, BL: blood island, PE: primitive endoderm. Scale bars: 500 μm. (E) E10.5 *Sox7*^*LacZ/WT*^ embryos. Left panel: S-gal staining on a section from dorsal aorta. Right: Immunostaining on the following section. Red arrows indicate emerging hematopoietic clusters. Yellow asterisks indicate SOX7::S-gal^+^ blood cells. Scale bars: 50 μm.

To define at the cellular level the expression of SOX7 during the earliest step of cardiovascular specification from mesoderm, we used an embryonic stem cell (ESC) line carrying a BAC transgene with the first exon of *Sox7* replaced by a *Gfp* reporter cDNA (Gandillet et al., 2009). These ESCs were differentiated *in vitro* to mesoderm *via* embryoid body (EB) formation (Fehling et al., 2003). This differentiation process led to the generation of a FLK1^+^ mesoderm population that was isolated and further differentiated to a TIE2^+^ cKIT^+^ cell population containing both hemogenic endothelial and EPCs as previously described (Lancrin et al., 2009). FLK1^+^ cells sorted from *Sox7-GFP* EBs were cultured as a monolayer and analysed after 2 days of culture (Fig. 1A). The SOX7-GFP^+^ fraction was strongly enriched for the expression of the endothelial marker TIE2, VE-Cadherin and CD31, and to a lesser extent, for c-KIT when compared to the SOX7-GFP^−^ fraction (Fig. 1B). Furthermore, the SOX7-GFP^+^ fraction had significantly higher transcript levels of *Flk1*, *Gata2*, and *Scl* genes, while there were also higher transcript levels of *Fli1* and *Cdh5 (*Fig. 1C). Collectively, these data indicate that *in vitro*, SOX7 is expressed in a very large fraction EPCs at the onset of endothelial specification from mesodermal precursors.

To investigate the pattern of SOX7 expression during *in vivo* development, mouse embryos heterozygous for a *Sox7-LacZ* null allele were generated. Whole mount S-gal staining of E7.5 embryos revealed the widespread presence of SOX7:LACZ-expressing cells in the yolk sac region of the developing conceptus in agreement with previously published data (Gandillet et al., 2009) (Fig. 1D). Further S-gal staining of E7.5 embryo sections confirmed the expression of SOX7 in the blood islands, allantois and primitive endoderm (Fig. 1D). In E10.5 embryos, S-gal staining highlighted SOX7::LACZ-expressing cells in the endothelium lining of the dorsal aorta (Fig. 1E). Additionally, immunostaining revealed that CD31^+^ c-KIT^+^ hematopoietic clusters expressed SOX7 (Fig. 1E, red arrows) whereas few hematopoietic cells within the aortal lumen also expressed SOX7 (Fig. 1E, yellow asterisks). These data confirm that SOX7 is expressed in the blood islands during the emergence of the first EPCs, as well as at later stages in endothelial cells during vascular development. It is interesting to note that SOX7 also appears to be expressed in hemogenic endothelium of the dorsal aorta since emerging clusters of blood cells do express SOX7. Given the very early onset of *Sox7* expression during the specification of the cardiovascular system, it is important to define how early during development this transcription factor is required for vasculogenesis and angiogenesis.

### 3.2 Sox7 complete knockout embryos have profound defects in vasculogenesis and angiogenesis

**Fig. 2:**
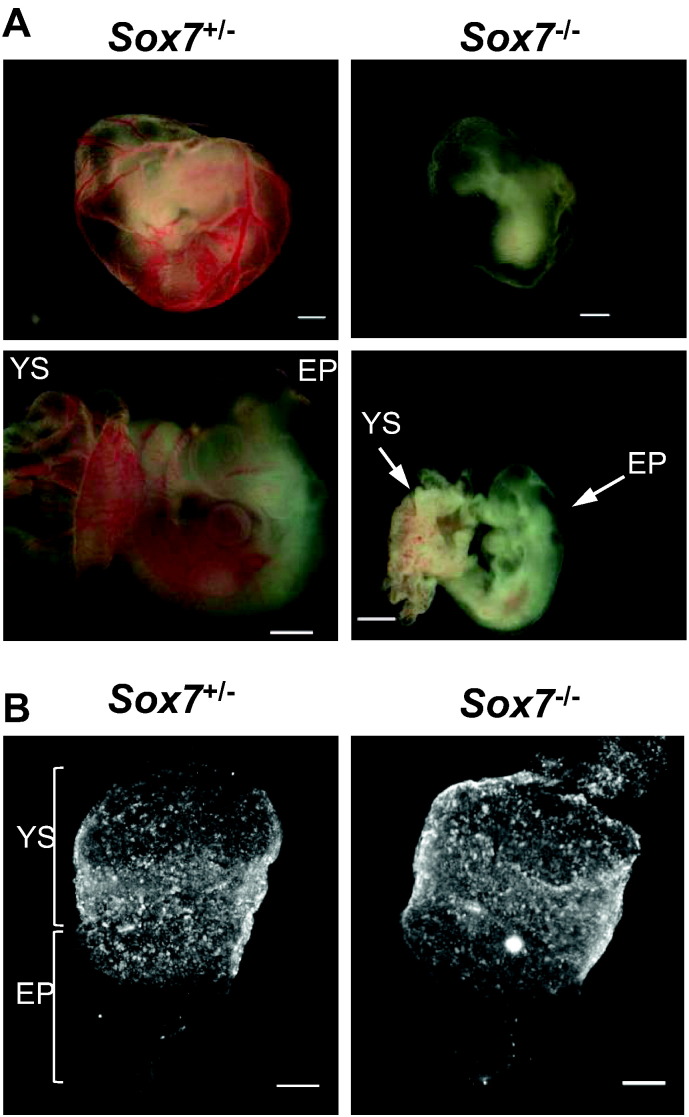
SOX7 deficient embryos show dramatic growth delay at E10.5 but generate primordial PECAM1^+^ cells at E7.5. (A) Light microscope images of heterozygous (*Sox7*^*+/−*^) and *Sox7* knockout (*Sox7*^*−/−*^) embryos at E10.5. Top panel: images of embryos embedded within their yolk sacs, bottom panel: images of embryo proper and yolk sac. (B) Whole mount PECAM1 staining of heterozygous (*Sox7*^*+/−*^) and *Sox7* knockout (*Sox7*^*−/−*^) yolk sacs embryos at E7.5. YS: yolk sac, EP: embryo proper.

**Fig. 3:**
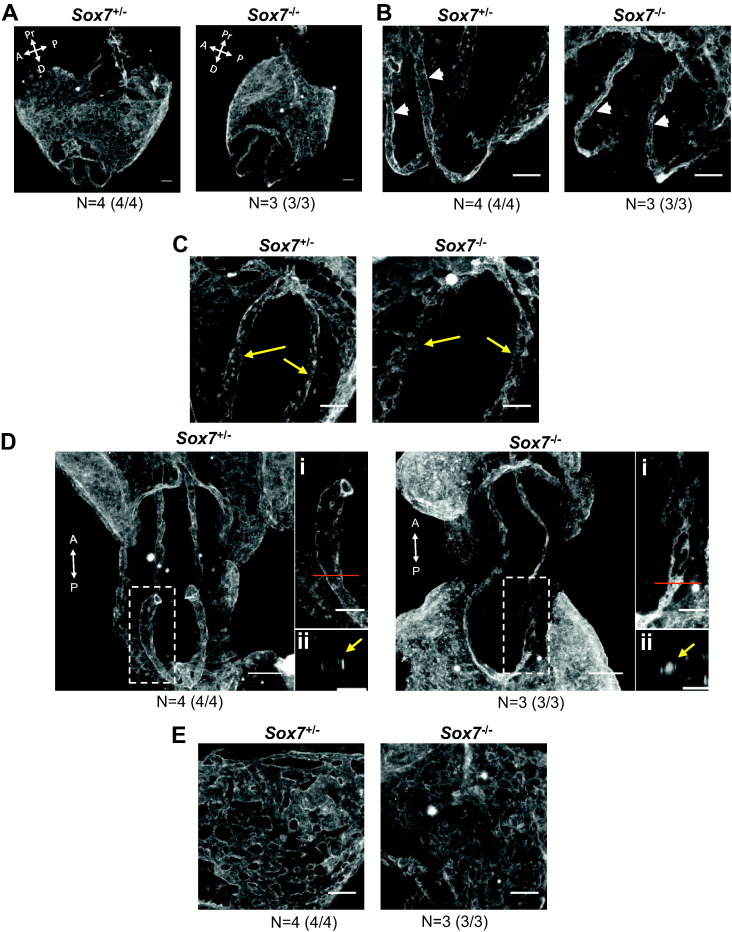
SOX7 deficient embryos show defects in the dorsal aorta and vascular plexus of the yolk sac at E8.5. (A) Whole mount PECAM1 staining of heterozygous (*Sox7*^*+/−*^) and *Sox7* knockout (*Sox7*^*−/−*^) embryos and yolk sacs at E8.5 (5–6 somite pairs). 3D projection of the embryo embedded within its yolk sac. White arrows indicate anterior (A), posterior (P), distal (D) and proximal (Pr) axes. (B) Anterior view of the dorsal aorta (white arrowheads). (C) Posterior tip of the dorsal aorta (yellow arrows). (D) Whole mount PECAM1 staining of *Sox7*^*+/−*^ and *Sox7*^*−/*−^ E8.5 embryos (3–5 somite pairs). The boxes denote area of magnification: (i) magnified view of dorsal aorta, red bar denotes cross section area, (ii) cross section of dorsal aorta lumen. (E) Details of the yolk sac vasculature. Scale bars: 100 μm. Data shown are representative of at least 3 embryos with 100% penetrance of the phenotype observed for knockout embryos.

In order to elucidate the role of *Sox7* during embryonic development, we first generated complete *Sox7* knockout embryos (*Sox7*^*−/−*^) on homogenous genetic background by backcrossing *Sox7*^*lacZ/+*^ mice on C57Bl/6 then by inter-crossing these transgenic mice. The LacZ cassette was inserted at the beginning of exon 2 and therefore fully disrupts the expression of *Sox7*. Complete deficiency in *Sox7* on this homogenous background led to a fully penetrant embryonic lethality phenotype by E10.5 characterised by severe growth retardation as well as an absence of large blood vessels in the yolk sac (Fig. 2A) as previously observed (Wat et al., 2012). To understand how these defects occurred, we investigated the formation of the vascular system prior to E10.5, a developmental time point at which *Sox7* deficiency resulted in lethality in all embryos examined. Whole mount PECAM1 staining at E7.5 revealed that *Sox7* deficiency did not affect the overall generation of PECAM1^+^ primordial EPCs (Fig. 2B). However, one day later by E8.5, *Sox7*^*−/−*^ embryos already displayed notable defects in the developing vascular networks which are formed by vasculogenesis (Fig. 3A). The development of the anterior region of the paired dorsal aorta was relatively unaffected (Fig. 3B, white arrowheads), but the posterior region displayed areas of highly unorganised endothelial cords rather than a distinct paired dorsal aorta (Fig. 3C, yellow arrows and Supplemental video 1). In addition, the posterior regions of the dorsal aorta were not lumenized in *Sox7*^−/−^ embryos (Fig. 3D). Finally, whilst a primitive vascular plexus formed within the yolk sac of *Sox7*^−/−^ embryos, the vascular network appeared disorganized compared to that of the control embryos (Fig. 3E).

**Fig. 4:**
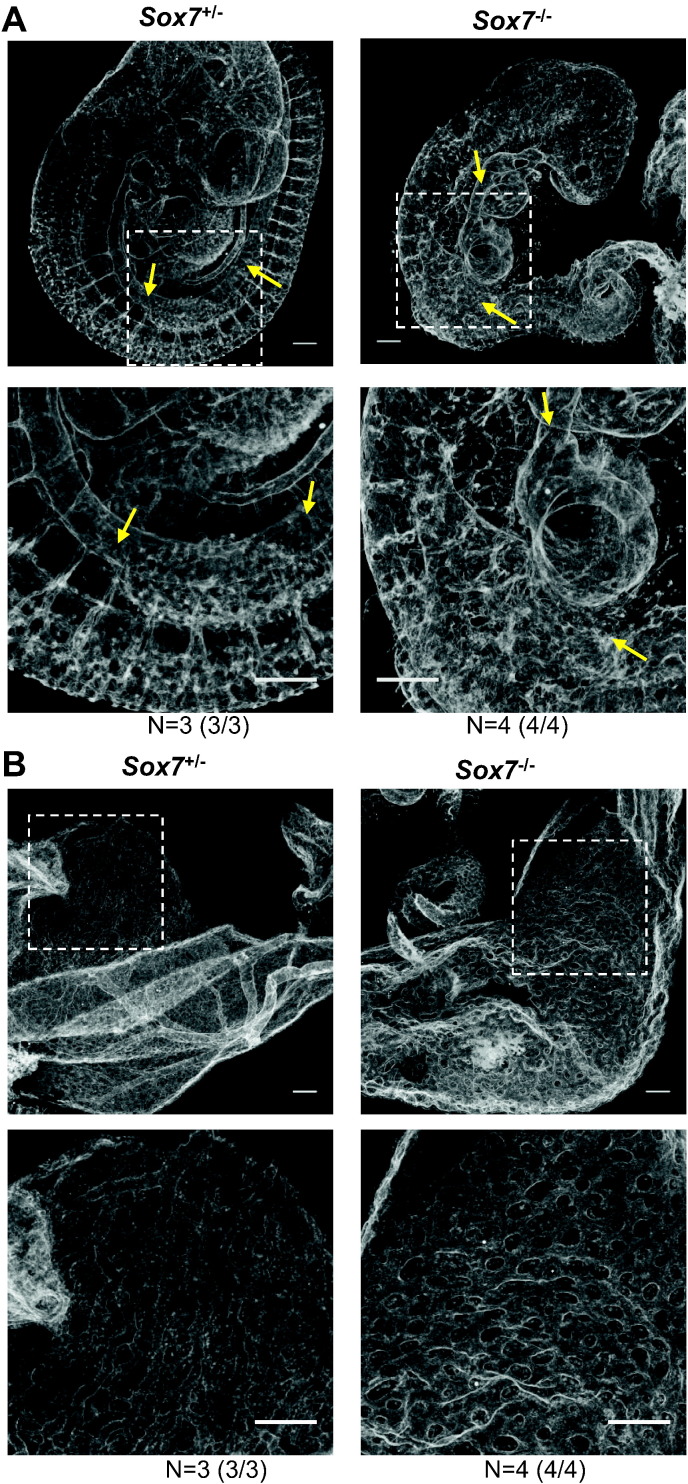
By E10.5 SOX7 deficient embryos have profound and widespread defects in vascular development. Whole mount PECAM1 staining of heterozygous (*Sox7*^+/−^) and *Sox7* knockout (*Sox7*^−/−^) embryos at E10.5. (A) Embryo proper, white boxes indicate areas of magnification. Yellow arrows indicate dorsal aorta. (B) Yolk sac vasculature, white boxes indicate areas of magnification. Scale bars: 250 μm.

By E10.5, whole mount PECAM1 staining revealed that *Sox7* deficiency led to extremely severe vascular defects both in the embryo proper and in the yolk sac (Fig. 4A and B). In *Sox7*^*−/−*^ embryos there was an absence of a definitive dorsal aorta in the posterior region of the embryo (Fig. 4A, yellow arrows), indicating that the dorsal aorta did not recover from the initial vasculogenic defects observed at E8.5. Furthermore, the highly unorganised nature of the vascular networks indicates considerable angiogenic remodelling defects resulting from *Sox7* deficiency. At E10.5, the yolk sac vasculature of *Sox7*^*−/−*^ embryos was arrested at the primitive vascular plexus stage, with a complete absence of vascular remodelling (Fig. 4B). Together these findings demonstrate that SOX7 is critically required for both vasculogenesis and vascular remodelling during angiogenesis. A detailed study of sprouting defect in the retina upon Cdh5-CreER induced deletion of *Sox7* has recently been published by Kim et al. (Kim et al., 2016), suggesting that SOX7 is important for both remodelling and sprouting during angiogenesis.

**Fig. 5:**
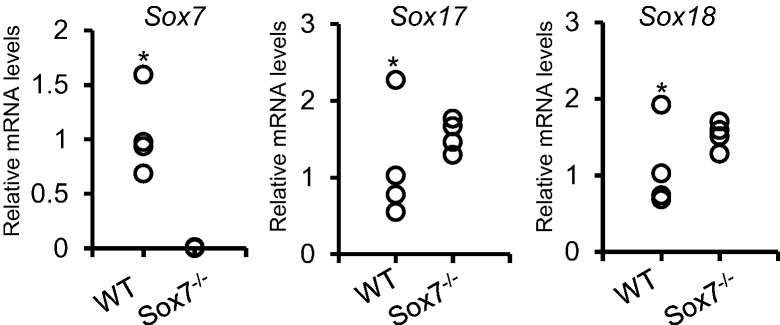
Change in the expression levels of *Sox17* and *Sox18* in SOX7 deficient embryos. PCR analysis of *Sox7*, *Sox17* and *Sox18* expression levels in wild type (WT) and *Sox7* knockout (*Sox7*^*−/−*^) embryos at E10 (n = 4).* Denotes the same WT embryo with overall higher levels of *Sox7*, *Sox17* and *Sox18*.

Unlike other intra-embryonic vessels and arteries, the nascent dorsal aortae originate from paired lateral cords that are formed by the migration and aggregation of angioblasts (Drake and Fleming, 2000, Sato, 2013). The fact that the posterior part of the dorsal aorta is affected in *Sox7* deficient embryos strongly suggests an early defect in cord assembly at a vasculogenesis stage. It has been well characterised that there is redundancy and compensation between SOXF family members in controlling vascular development (Hosking et al., 2009, Matsui et al., 2006, Zhou et al., 2015). The conditional knockout of SOX7 in TIE2 expressing endothelial cells using mice from a mixed genetic background, resulted in relatively minor vascular defects such as a decrease in the diameter of the dorsal aorta (Zhou et al., 2015). These relatively minor defects reported by Zhou and collaborators are most likely due to compensation by SOX17 and SOX18 rather than truly a result of *Sox7* deletion in TIE2-expressing cells. Indeed, it was shown that in *Sox18*^*−/−*^ mice of a mixed genetic background SOX7 and SOX17 were upregulated and substituted for SOX18 (Hosking et al., 2009). Given this known redundancy and compensation among the three SOXF genes, we examined transcript levels for *Sox17* and *Sox18* in *Sox7*^*−/−*^ embryos relative to *Sox7*^*+/+*^ embryos (Fig. 5). This analysis revealed an increase in the expression of both *Sox17* and *Sox18*, suggesting possible compensation of SOX7 deficiency by SOX17 and SOX18. However, even with the compensatory activity by SOX17 and SOX18, *Sox7* deficiency resulted in massive vasculogenic and angiogenic defects. Together these data support a critical and unique role for SOX7 in the development of the vascular system.

### 3.3 Sox7 conditional deletion in FLK1-expressing cells leads to severe defects in vasculogenesis and angiogenesis

**Fig. 6:**
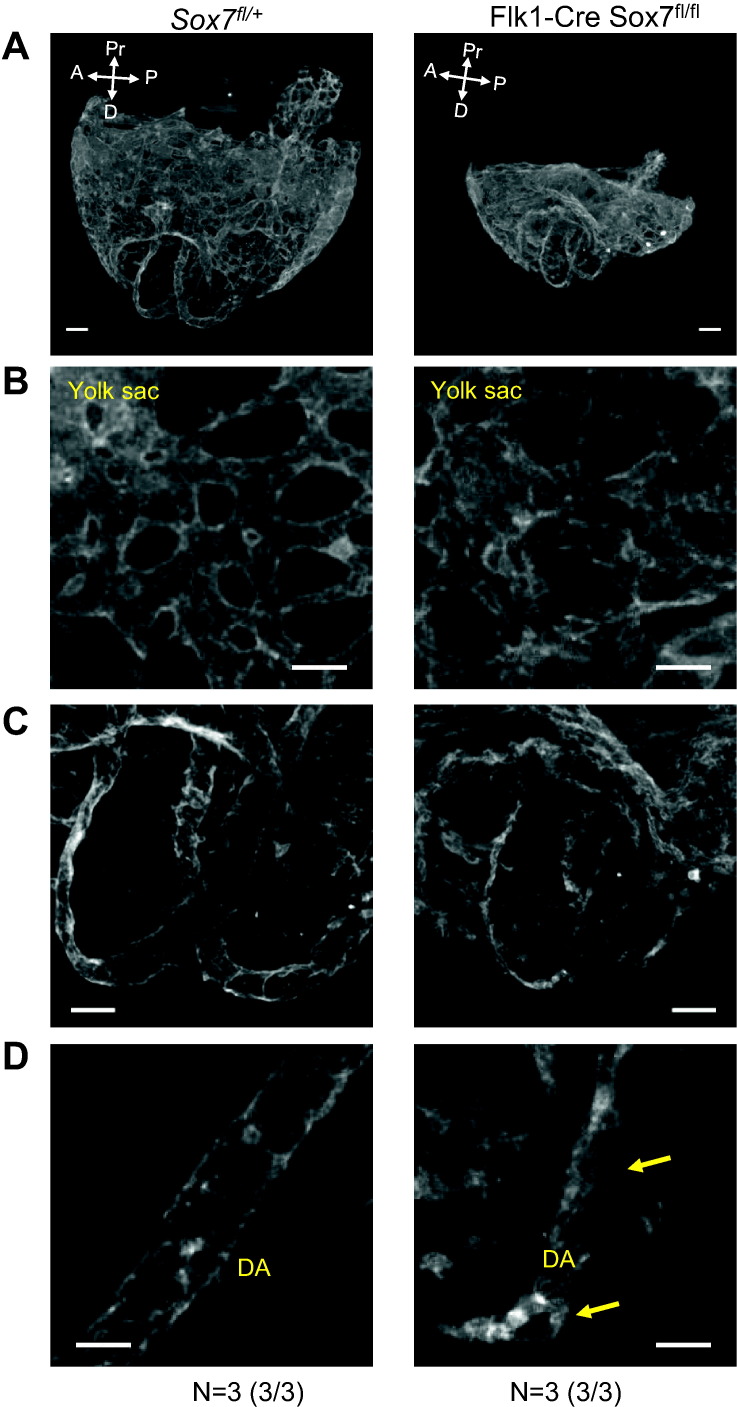
*Flk1*-*Cre Sox7*^*fl/f*l^ embryos have defects in the dorsal aorta at E8.5. Whole mount PECAM1 staining of control *(Sox7*^fl/+^) and *Flk1*-*Cre Sox7*^*fl/f*l^ embryos and yolk sac at E8.5 (5–7 somite pairs). (A) 3D projection of embryos embedded within their yolk sacs, white arrows indicated anterior (A), posterior (P), distal (D) and proximal (Pr) axes. (B) Details of the yolk sac vasculature. (C) Paired dorsal aorta region. (D) Magnified view of a posterior region of a single dorsal aorta. Yellow arrows indicate malformation in the dorsal aorta. Scale bars 100 μm (A and C), 50 μm (B and D). Data are representative of at least three embryos with 100% penetrance of the phenotype observed for knockout embryos.

**Fig. 7:**
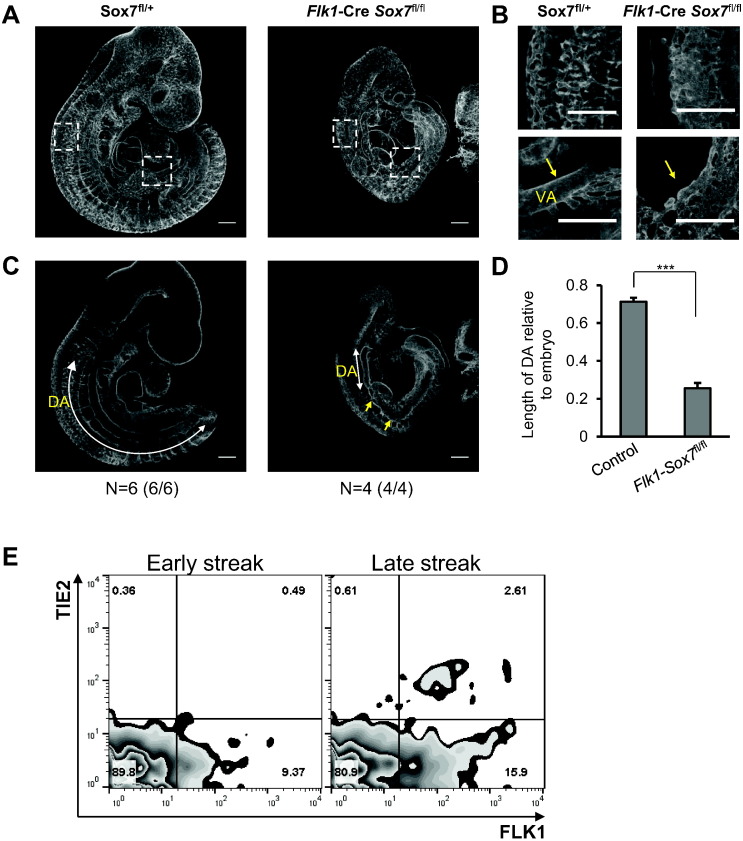
*Flk1*-*Cre Sox7*^*fl/f*l^ embryos have severe and widespread vascular defects at E10.5. Whole mount PECAM1 staining of *Sox7*^*fl/+*^ and *Flk1*-*Cre Sox7*^*fl/f*l^ embryos at E10.5. (A) 3D projection of embryo proper, white boxes indicate areas of magnification. Data shown are representative of 4 embryos. (B) Top panel: organization of capillaries in posterior region, bottom panel: vitelline artery (VA). (C) Sagittal slices through the embryo proper. DA: dorsal aorta, white arrow indicates length of functional dorsal aorta; yellow arrows indicate malformation of the DA. (D) Mean length of DA relative to embryo ± SEM, n = 6 control (*Sox7*^*fl/+*^ and *Sox7*^*fl/fl*^) *versus* n = 4 *Flk1*-*Cre Sox7*^*fl/f*l^ embryos. (E) Expression of FLK1 and TIE2 in wild type gastrulating embryos at early streak (left panel) and late streak (right panel) stages measured by flow cytometry.

In addition to its expression in endothelial progenitors, *Sox7* has been previously detected in primitive endoderm (Futaki et al., 2004, Murakami et al., 2004), in the earliest specified hematopoietic progenitors (Gandillet et al., 2009) and in emerging hematopoietic clusters in the dorsal aorta (Lilly et al., 2016, Nobuhisa et al., 2014). To define a possible role and requirement for *Sox7* in specific compartments, we generated a *Sox7* conditional allele in which the exon 2 of *Sox7* is flanked by LoxP sequences and can be excised upon CRE expression. First, we analysed the requirement for *Sox7* expression in the vascular compartment using a *Flk1-cre* transgenic mouse line (Motoike et al., 2003). The conditional deletion of *Sox7* in FLK1-expressing cells resulted in early embryonic lethality and a phenotype very similar to the complete *Sox7* knockout embryos. At E8.5, whole mount PECAM1 staining revealed that *Flk1-Cre Sox7*^*fl/f*l^ embryos displayed already noticeable defects in the developing vascular networks (Fig. 6A–D) including a disorganized yolk sac vascular plexus (Fig. 6B) and areas of highly unorganised endothelial cords in the posterior region of the dorsal aorta (Fig. 6C–D, yellow arrows). By E10.5, the highly unorganised nature of the vascular networks was indicative of considerable angiogenic defects resulting from the deletion of *Sox7* in FLK1-expresssing cells (Fig. 7A) in agreement with the recently published phenotype of the *Tie2* specific *Sox7* knockout embryos (Kim et al., 2016) that was performed on homogenous genetic background unlike the study by Zhou and collaborators (Zhou et al., 2015). In contrast to *Tie2-*specific *Sox7* deletion, the endothelial *Flk1*-specific deletion of *Sox7* revealed marked defects in the formation of the major blood vessels in the embryo indicative of vasculogenic defects. In *Flk1*-*Cre Sox7*^*fl/f*l^ embryos, there was a lack of an observable vitelline artery in the embryo proper (Fig. 7B, yellow arrows). Furthermore, the functional dorsal aorta in *Flk1*-*Cre Sox7*^*fl/f*l^ embryos was extremely short, with the posterior region of the dorsal aorta resembling a cord of endothelial cells suggesting major defects in the vasculogenic events leading to the formation and organization of the angioblast cords giving rise to the posterior region of the dorsal aorta (Fig. 7C–D, white arrows). The lack of vitelline artery is most likely a direct consequence of the absence of the posterior dorsal aorta as the vitelline artery arises from the dorsal aorta (Drake and Fleming, 2000). It is likely that the differences between the *Sox7 Tie2*-deleted and *Flk1*-deleted embryonic phenotypes results from the earlier expression of FLK1 during development (Fig. 7E) and therefore the earlier deletion of Sox7 in *Flk1-cre* than in *Tie2-cre* embryos. Unlike *Tie2*, the expression of *Flk1* is detected in mesoderm and mesenchyme (Ema et al., 2006), it is possible that *Sox7* deletion in these tissues contributes to the stronger phenotype observed. These findings demonstrate that the expression of SOX7 is required earlier than previously described (Kim et al., 2016) and that SOX7 is an important transcriptional regulator of vasculogenesis.

**Fig. 8:**
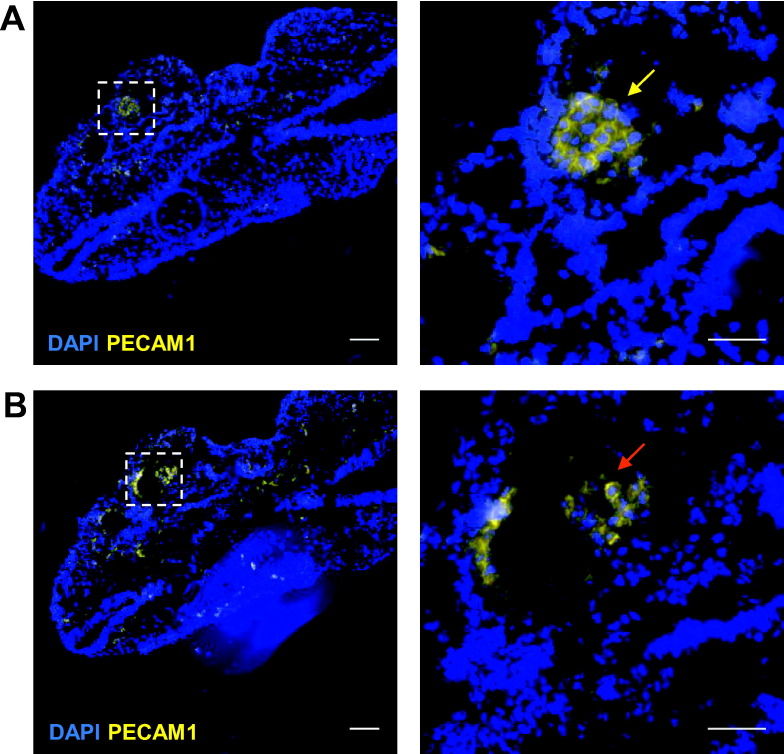
*Flk1*-*Cre Sox7*^*fl/f*l^ embryos have lumenization defects in blood vessels. (A-B) PECAM1 staining of E9.5 *Flk1*-*Cre Sox7*^*fl/f*l^ embryo sections. Left panels: 10 × objective, scale bars: 100 μm; right panels: 40 × objective, scale bars: 50 μm. Boxes denote areas of magnification. Top and bottom panels are two subsequent sections of the embryo. Yellow arrow: nonlumenized section of a blood vessel, red arrow: lumenized section of the same blood vessel.

**Fig. 9:**
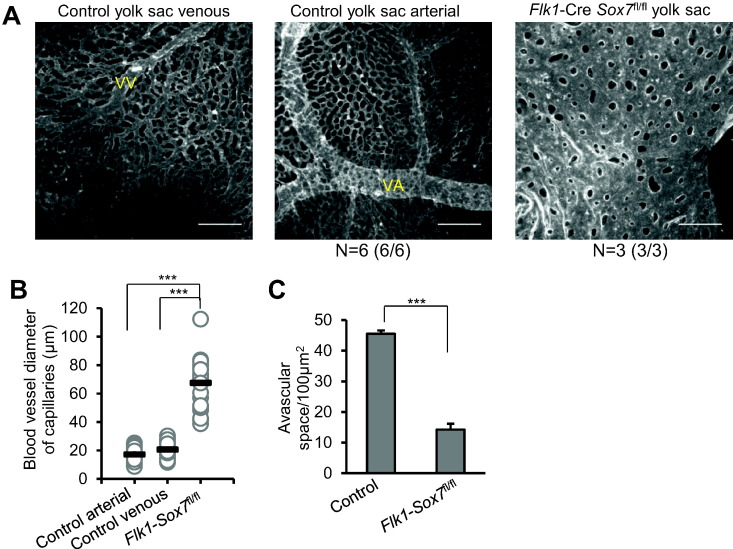
Complete absence of remodelling in the yolk sac of *Flk1*-*Cre Sox7*^*fl/f*l^ embryos. (A) Flat mounted E10.5 yolk sacs stained for PECAM1. Control yolk sacs have venous and arterial systems and large blood vessels such as a vitelline vein (VV) and a vitelline artery (VA). Data shown are representative of 3 embryos. (B) Diameter of capillaries in a control yolk sac (*Sox7*^*fl/+*^), compared with a *Flk1*-*Cre Sox7*^*fl/fl*^ yolk sac. Data are presented as mean diameter (black bar), n = 30 capillaries. (C) Avascular space of capillaries/100μm^2^ in control *versus Flk1*-*Cre Sox7*^*fl/fl*^ yolk sacs. Data are presented as mean ± SEM, n = 6 control and n = 3 *Flk1*-*Cre Sox7*^*fl/f*l^ yolk sacs. All statistical analyses are Student's two-tailed *t*-test. Scale bars = 500 μm.

To further analyse the vascular defects in *Flk1*-*Cre Sox7*^*fl/f*l^ embryos, we performed PECAM1 staining on sections of E9.5 embryos (Fig. 8A and B). The complete disorganisation of the vascular network made comparisons of specific blood vessels with control embryos impossible. However, there was clear evidence of lumenization defects in a major blood vessel, as a mass of disorganized endothelial cells was observed (Fig. 8A, yellow arrow) that in the subsequent section formed a blood vessel with a lumen (Fig. 8B, red arrow). At E10.5 the yolk sac vasculature of *Flk1*-*Cre Sox7*^*fl/f*l^ embryos was arrested at a primitive vascular plexus stage, with a complete absence of vascular remodelling (Fig. 9A). In control yolk sacs, venous and arterial areas were easily identified along with the vitelline vein (VV) and vitelline artery (VA); in contrast the yolk sac of *Flk1*-*Cre Sox7*^*fl/f*l^ embryos only contained a homogenous plexus of vessels with relatively large diameters as shown by blood vessel diameter measurement (Fig. 9B). Furthermore, measurement of the space between capillaries identified that *Flk1*-*Cre Sox7*^*fl/f*l^ plexuses have a decreased avascular space compared to capillaries of control yolk sacs (Fig. 9C). This is in contrast to embryos with hemodynamic flow deficiencies which show larger avascular space between non-remodelled yolk sac plexus blood vessels when compared to control embryos (Jones et al., 2008). Together, this suggests that the phenotype of the *Flk1*-*Cre Sox7*^*fl/f*l^ embryos is largely endothelial based and not due to cardiac defects causing decreased blood flow. Taken together, these data demonstrate that SOX7 is critically required in FLK1-expressing cells for both vasculogenesis and angiogenesis. In particular, SOX7 seems to be critical for the formation of a fully lumenized dorsal aorta, suggestive of an incomplete circulatory loop: a phenotype similar to that of SOX7^−/−^ zebrafish (Hermkens et al., 2015). However, unlike the zebrafish model, SOX7 deficiency in the mouse embryo also results in angiogenesis defects as demonstrated by the absence of remodelling and unorganised patterning of the entire vascular network.

The conditional deletion of *Sox17* in TIE2-expressing cells also resulted in 100% embryonic lethality by E12.5 with major alterations in vascular remodelling, in the development of large arteries and in sprouting angiogenesis (Corada et al., 2013, Lee et al., 2014). *Sox17* was also implicated in the acquisition and maintenance of arterial identity (Corada et al., 2013), while this role was shown to be performed by *Sox7* in Zebrafish (Hermkens et al., 2015). It should be noted however that in Zebrafish *Sox17* lacks the β-catenin binding domain which might explain these differences between mouse and zebrafish models (Chung et al., 2011). Together, these data suggested that both *Sox7* and *Sox17* are essential for the development of the vascular system and that they cannot compensate for each other, at least on homogeneous genetic background, and that they have slightly different roles in vasculogenesis and arterial specification, in line with their distinctive pattern of expression (Zhou et al., 2015).

Our findings also establish that while SOX7 is expressed in primitive endoderm (Murakami et al., 2004, Niimi et al., 2004), SOX7 is dispensable for the formation, maintenance or function of this lineage. Indeed, we observed an identical phenotype in *Sox7* complete knockout embryos in which Sox7 is deleted in primitive endoderm and FLK1-specific *Sox7* deficient embryos in which Sox7 is not deleted in primitive endoderm. Together this suggests that SOX7 deficiency in primitive endoderm in the complete knockout does not affect the early steps of embryonic development in which primitive endoderm plays a critical role in body plan formation and tissue induction (Stern and Downs, 2012). It is possible however that in *Sox7* complete knockout embryos SOX17 compensates for SOX7 loss in primitive endoderm since SOX17 is also expressed in this lineage (Engert et al., 2009, Niimi et al., 2004). The generation of *Sox7* and *Sox17* double knockout embryos would be required to address the specific role of SOXF factors in primitive endoderm formation and function.

### 3.4 Sox7 conditional deletion in VAV-expressing cells does not affect the hematopoietic system

**Fig. 10:**
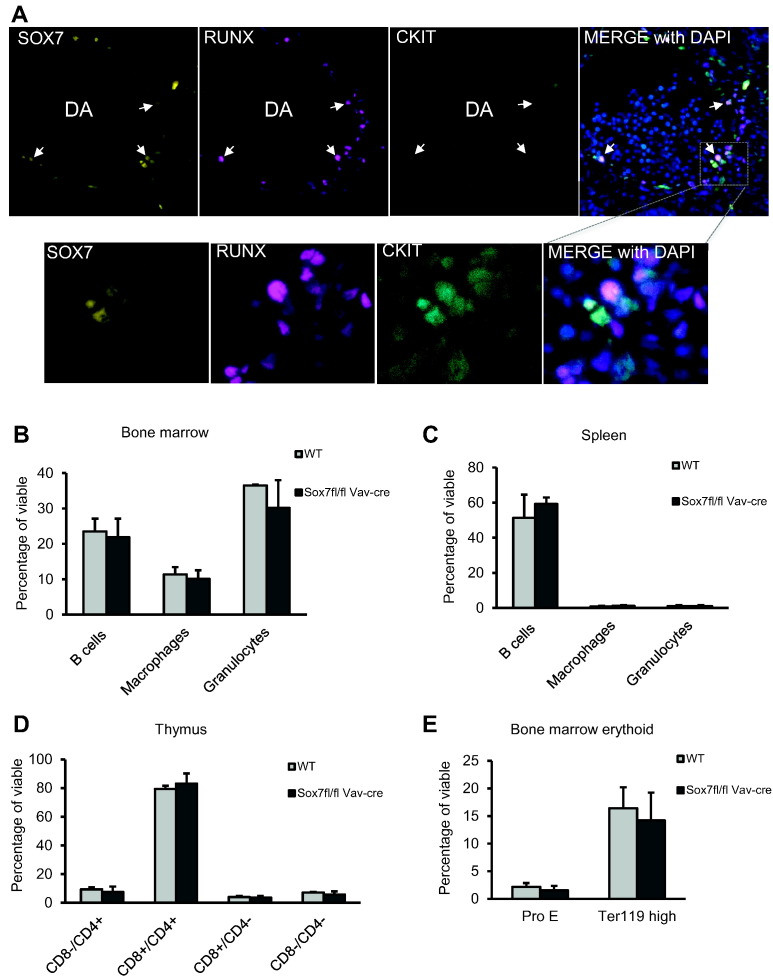
*Vav-Cre Sox7*^*fl/fl*^ mice live to adulthood and have no hematopoietic defects. (A) Immuno-staining of E10.5 wild embryo transversal section. White arrows indicate example of cells co-expressing SOX7, RUNX and cKIT. Upper panels are 40 × magnification; lower panels are 100 × magnification. DA: dorsal aorta. (B and E) Bone marrow, (C) spleen and (D) thymus harvested from aged matched adult WT and *Vav-Cre Sox7*^*fl/fl*^ mice and analysed by flow cytometry, gating first on the viable cells. B cells: CD19^+^ and/or B220^+^; macrophages: CD11b^+^ and F4/80^+^; granulocytes: CD11b^+^ and GR1^+^; Pro erythroblast (Pro E): Ter119^med^ and CD71^+^; maturing erythroid cells: Ter119^high^. Data are presented as mean ± SE, n = 3 mice in each group. No differences were observed between WT and *Vav-Cre Sox7*^*fl/f*l^ populations (Student's paired two-tailed *t*-test).

Finally, we analysed the consequence of the specific deletion of *Sox7* in the hematopoietic compartment given the observed expression of SOX7 at all sites of hematopoietic emergence during embryogenesis (Lilly et al., 2016). Indeed, this was further confirmed by the co-expression of SOX7 with RUNX and cKIT, both marking emerging blood cells in the ventral aspect of the dorsal aorta in wild type embryos at E10.5 (Fig. 10A). To determine a possible role of SOX7 in hematopoiesis, *Sox7*^*fl/fl*^ mice were crossed with *Vav-Cre* transgenic mice (de Boer et al., 2003), which resulted in *Sox7* deficiency in all definitive hematopoietic cells. Interestingly, *Vav-Cre Sox7*^*fl/f*l^ pups were viable and lived to adulthood without any phenotypic abnormalities or observable defects in the hematopoietic system (Fig. 10B–E). These data demonstrate that while *Sox7* is expressed in the earliest blood progenitors, this transcription factor is not required for definitive hematopoiesis which encompasses all blood cells generated from E8.5 onward, including hematopoietic stem cells. However, it remains possible that the two other SOXF factors are providing enough compensation to allow for the emergence of blood cells in developing embryos deficient for *Sox7* expression in *Vav*-expressing cells. The generation of triple *SoxF* conditional embryos would be required to address this possibility.

## 4 Concluding remark

The development of new vascular networks *via* vasculogenesis and angiogenesis is an important factor in the pathophysiology of all solid tumours. Neoplastic vascularisation facilitates the proliferation and subsequent metastasis of tumour cells, making angiogenic processes attractive targets in combating cancer (Nishida et al., 2006). The role of SOX7 in promoting tumour progression and angiogenesis is poorly understood. Recent data suggest that SOX17 is an important regulator of tumour angiogenesis (Yang et al., 2013). Together, these findings warrant further investigation into whether SOXF factors act redundantly or compensate for each other to promote tumour angiogenesis, which may offer novel therapeutic targets for the treatment of cancer.

## Conflict of interest

The authors declare no competing financial interests.

The following are the supplementary data related to this article.

Supplemental video 1 *Sox7*^−/−^ embryos have defects in the dorsal aorta at E8.5. Whole mount PECAM1 staining of E8.5 *Sox7*^+/−^ and *Sox7*^−/−^ embryos. Arrows indicate posterior section of dorsal aorta. Supplemental video 1
