# Supplementary figures and images for: SOX7 expression is critically required in FLK1-expressing cells for vasculogenesis and angiogenesis during mouse embryonic development
Source: Mech Dev. 2017 Aug;146:31–41. doi: 10.1016/j.mod.2017.05.004 (PMC5496588; doi:10.1016/j.mod.2017.05.004)

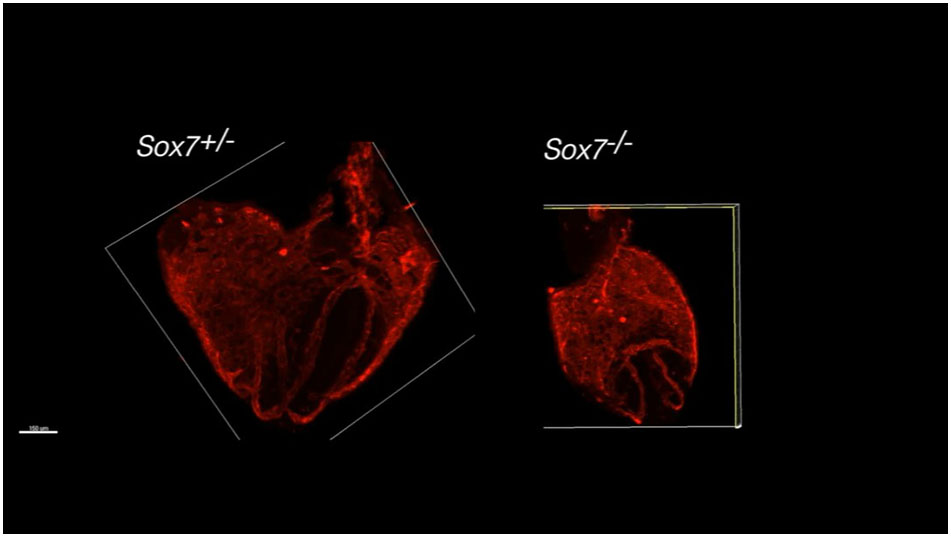

Supplement: Supplemental video 1 — Sox7−/− embryos have defects in the dorsal aorta at E8.5. Whole mount PECAM1 staining of E8.5 Sox7+/− and Sox7−/− embryos. Arrows indicate posterior section of dorsal aorta. [file mmc1.jpg]
